# Assessment of histological characteristics, imaging markers, and rt-PA susceptibility of ex vivo venous thrombi

**DOI:** 10.1038/s41598-021-02030-7

**Published:** 2021-11-23

**Authors:** Samuel A. Hendley, Alexey Dimov, Aarushi Bhargava, Erin Snoddy, Daniel Mansour, Rana O. Afifi, Geoffrey D. Wool, Yuanyuan Zha, Steffen Sammet, Zheng Feng Lu, Osman Ahmed, Jonathan D. Paul, Kenneth B. Bader

**Affiliations:** 1 Committee on Medical Physics, University of Chicago, Chicago, IL 60637 USA; 2 Department of Radiology, Weill Cornell Medicine, New York, NY 10021 USA; 3 Department of Radiology, University of Chicago, Chicago, IL 60637 USA; 4 Department of Cardiothoracic and Vascular Surgery, University of Texas at Houston, Houston, TX 77030 USA; 5 Department of Pathology, University of Chicago, Chicago, IL 60637 USA; 6 The Human Immunological Monitoring Facility, University of Chicago, Chicago, IL 60637 USA; 7 Department of Medicine, University of Chicago, Chicago, IL 60637 USA

**Keywords:** Cardiovascular biology, Predictive markers

## Abstract

Venous thromboembolism is a significant source of morbidity and mortality worldwide. Catheter-directed thrombolytics is the primary treatment used to relieve critical obstructions, though its efficacy varies based on the thrombus composition. Non-responsive portions of the specimen often remain in situ*,* which prohibits mechanistic investigation of lytic resistance or the development of diagnostic indicators for treatment outcomes. In this study, thrombus samples extracted from venous thromboembolism patients were analyzed ex vivo to determine their histological properties, susceptibility to lytic therapy, and imaging characteristics. A wide range of thrombus morphologies were observed, with a dependence on age and etymology of the specimen. Fibrinolytic inhibitors including PAI-1, alpha 2-antiplasmin, and TAFI were present in samples, which may contribute to the response venous thrombi to catheter-directed thrombolytics. Finally, a weak but significant correlation was observed between the response of the sample to lytic drug and its magnetic microstructure assessed with a quantitative MRI sequence. These findings highlight the myriad of changes in venous thrombi that may promote lytic resistance, and imaging metrics that correlate with treatment outcomes.

## Introduction

Venous thromboembolism (VTE) is a major health problem that affects 600,000 Americans annually^1^, and up to 10 M worldwide^2^. There is a significant financial burden, with healthcare costs exceeding seven billion dollars each year in the United States associated with VTE^3^. The two primary clinical manifestations of VTE are pulmonary embolism (PE) and deep vein thrombosis (DVT). Pulmonary embolism is the most serious manifestation of VTE, and carries a 25% mortality rate and a 30-day survival rate of 59%^4^. It is estimated that 80% of PE cases originate from deep vein thrombi, primarily in the iliofemoral vasculature^5^. Beside the potential for embolization, there is prevalent morbidity associated with DVT including chronic painful leg swelling and/or venous ulcerations^6^. Phlegmasia cerulea dolens is another disease state secondary to DVT that may result in compromised circulation^7,8^. Rapid restoration of flow is required for phlegmasia cerulea dolens patients to prevent amputation of the afflicted limb (15%) or death (25%)^9,10^. While not as widely studied as arterial thrombosis, these data illustrate VTE requires effective treatments and screening methods.

Anticoagulation therapy alleviates the symptoms of VTE and prevents thrombus growth^11^. Heparin is administered in the acute settings as an antithrombotic, followed by direct oral anticoagulants to inhibit factor Xa or thrombin^12,13^. More aggressive approaches are often necessary to prevent the most egregious outcomes of DVT and PE. The standard of care for large vessel recanalization is catheter-directed thrombolytics, which is administered over the course of hours to days^14^. Recombinant tissue plasminogen activator (rt-PA) is the principle thrombolytic drug used in the western world^15^. This approach disintegrates fibrin components within the thrombus, and is effective for acute disease^16–19^. Chronic thrombosis, defined as a single thrombus that persists for more than seven days, is a prevalent feature of VTE^20,21^ and is resistant to rt-PA^22^. Better long-term patient outcomes are observed for when the vessel is fully recanalized^23^, and lytic can be administered up to four days in an attempt to disintegrate residual thrombus^14^. The extended treatment time increases the risk of serious bleeding complications associated with rt-PA^24^ and healthcare costs. Alternative interventions such as mechanical extraction^25,26^ or histotripsy-aided thrombolysis^27^ can be effective for thrombi non-responsive to lytics. The development of methods to identify lytic-resistant, chronic thrombi a priori will expedite the use of these alternative treatment schemes or new approaches for chronic VTE strategies.

The precise histologic properties of chronic VTE remains elusive. Animal models do not fully replicate the clotting cascade of humans^28–31^, and traditional treatment methods leave the sample in situ^32^. The advent of venous-specific thrombectomy devices enables access to VTE samples for characterization^33–35^. A goal of this study was to conduct histological analysis on VTE specimen ex vivo. Histology provides semi-quantitative assessment of tissue structure, and remains the foundation for pathological analysis^36^. Whole sample imaging markers of iron (marker of erythrocytes) via quantitative MRI susceptibility mapping and stiffness (marker of extracellular thrombus structure) via ultrasound shear-wave elastography were collected. Finally, an in vitro assay was conducted to determine the lytic susceptibility of the specimen. Primary findings indicate that collagen increased and erythrocytes decreased with thrombus age, and the presence of the fibrinolysis inhibitors plasminogen activator inhibitor 1, alpha 2-antiplasmin, and thrombin-activatable fibrinolysis inhibitor in samples. Further, the lytic susceptibility of thrombus correlated with its magnetic susceptibility assessed via MRI. The observed changes in clot structure reflect the complex mechanisms by which VTE microstructure may develop lytic resistance, and these mechanisms can be gauged using imaging metrics.

## Results

### Approach

Analysis was performed on 26 venous thrombi aspirated acutely (12 deep vein thrombi and 14 pulmonary emboli). Details for the patient demographics are presented in Supplemental Table 1. Each sample was sectioned into up to five segments. Three segments ~ 0.5 cm in length were subjected to pathological analysis (histology, immunohistochemistry, and immunofluorescence), one segment ~ 2 cm in length was subjected to imaging analysis (MRI and ultrasound), and one segment ~ 1 cm in length was used to test rt-PA susceptibility. Not all samples were large enough to conduct each set of analyses (Histology: 26 samples, Lytic susceptibility: 9 samples, Imaging analysis: 9 for MRI and 12 for ultrasound). For histological studies, specimen segments were stained to highlight erythrocytes, fibrin, collagen, and platelets for a total of 234 total images analyzed. Multiplexed immunofluorescence was used to assess the co-location of the thrombus components identified with histology and immunohistochemistry. For MRI and ultrasound imaging analysis, the specimen segments were embedded in agarose gel. Ultrasound shear wave elastography was used to assess the elastic modulus of the sample, and the MRI sequence quantitative susceptibility mapping to gauge the fraction of erythrocytes in the sample. Finally, an in vitro assay was conducted to determine lytic susceptibility by exposing the specimen segment to plasma and rt-PA. Further information on processing samples for each type of analysis is summarized in the methods section.

### Histological assessment of thrombus

**Figure 1:**
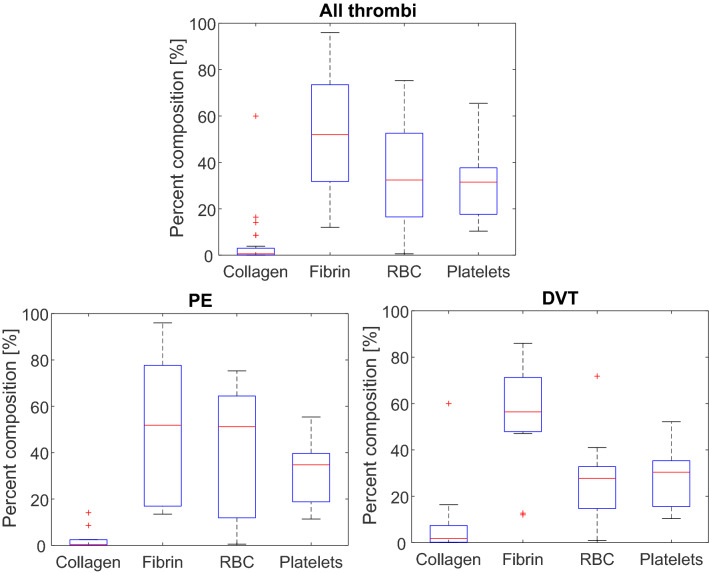
The fractional area of collagen, fibrin, platelets, and erythrocytes (RBC) present in (top) all analyzed VTE specimen (26 samples), (bottom left) pulmonary emboli (14 samples), and (bottom right) deep vein thrombi (12 samples). Components were assessed using histochemical staining. Colorimetric analysis was conducted in ImageScope (Leica, Biosystems, Germany) to identify pixels associated with each components. Horizontal red lines indicate median values. The top and bottom portions of the blue box represent the 25th and 75th percentiles, respectively, and whiskers extent to the data points not considered outliers. Red crosses indicate outliers, which correspond to datapoints greater than ${{q}}_{75} + 1.5 \times \left( {{{q}}_{75} - {{q}}_{25} } \right)$ or less than ${{q}}_{25} - 1.5 \times \left( {{{q}}_{75} - {{q}}_{25} } \right)$, where ${{q}}_{25}$ corresponds to the 25 th percentile of the sample data, and ${{q}}_{75}$ corresponds 75th percentile of the sample data^37^.

**Figure 2:**
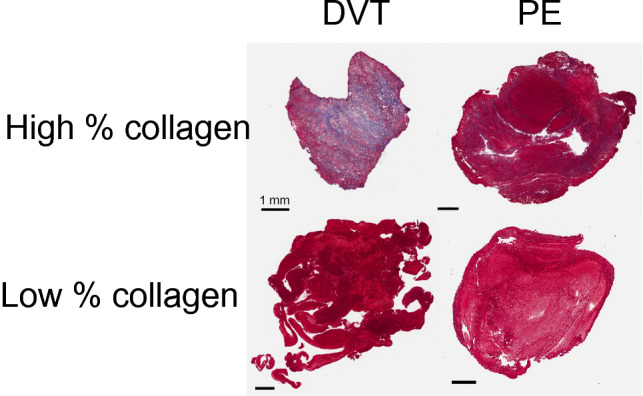
Observed patient-to-patient variability in collagen observed in ex vivo thrombus samples. Deep vein thrombi (DVT) are shown in the left column, pulmonary emboli (PE) are shown in the right column. Collagen appears blue in the Masson’s trichrome stain and compromises 60% (top left) and less than 1% (bottom left) for DVT, and 14% (top right) and less than 1% (bottom right) for PE by area. Colorimetric analysis was conducted in ImageScope (Leica Biosystems, Germany) to identify pixels associated with collagen. Color thresholds were evaluated and accepted by a board-approved pathologist. The scale bars indicate 1 mm.

The fraction of fibrin, collagen, erythrocytes, and platelets observed in samples is shown in Fig. 1. A wide range in composition was noted, with fibrin and erythrocytes as the primary thrombus components (52 ± 26% and 35 ± 23% of thrombus area, respectively). Collagen constituted less than 2% of the thrombus area on average, though a maximum of ~ 60% was observed in one sample (Fig. 2). When present, collagen was observed in discrete clusters those prevalence increased towards the edge of the thrombus (i.e. towards the thrombus/vessel wall interface). Platelets were observed as a prevalent feature as well in samples, and their density also increased towards the edges of the sample. Erythrocytes tended to be located near the center of the thrombus and were less likely to be observed along the edges. Fibrin was uniform across the samples.

Analysis was also conducted to categorize the samples based on anatomic location of extraction (Fig. 1). Mean values for the area of collagen, fibrin, erythrocytes, and platelets were not significantly different between PE and DVT samples. However, the sample-to-sample variability in fibrin and erythrocytes was reduced in DVT samples in comparison to PE.

**Figure 3:**
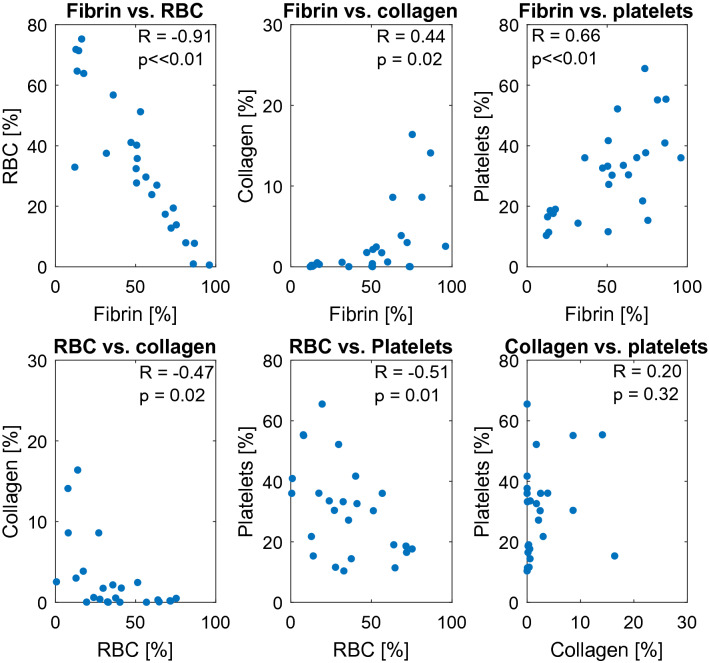
Trends in thrombus composition for fibrin, collagen, red blood cells (RBCs), and platelets for all samples (N = 26 total). The Pearson’s correlation coefficient (R) and p-values are reported for each respective pair.

Trends between collagen, fibrin, erythrocytes, and platelets are shown in Fig. 3 to gauge the interdependence of thrombus components using Pearson’s correlation coefficient, R (N = 26 samples). The strongest correlation was a negative relationship between fibrin and erythrocytes (R = − 0.91; *p* < 0.01). A medium-strength positive correlation was observed between fibrin and platelets (R = 0.66; *p* < 0.01). Significant correlations were also noted between erythrocytes and platelets (negative) (R = − 0.51; *p* = 0.01), erythrocytes and collagen (positive) (R = 0.47; *p* = 0.02), and fibrin and collagen (positive) (R = 0.44; *p* = 0.02). There was no correlation observed between collagen and platelets (R = 0.20; *p* = 0.3).

### Change in histological properties with thrombus age

**Figure 4:**
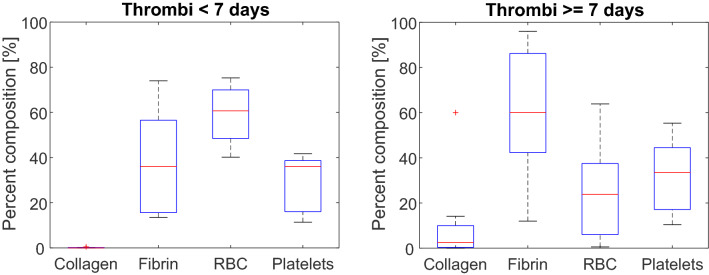
Histological analysis for acute (less than 7 days old, N = 12) and chronic (greater than or equal to 7 days old, N = 14) thrombi. None of the 26 samples were excluded from analysis. Red crosses indicate outliers and horizontal red lines indicate median values. The top and bottom portions of the blue box represent the 25th and 75th percentiles, respectively, and whiskers extent to the data points not considered outliers. Outliers correspond to datapoints greater than $q_{75} + 1.5 \times \left( {q_{75} - q_{25} } \right)$ or less than $q_{25} - 1.5 \times \left( {q_{75} - q_{25} } \right)$, where $q_{25}$ corresponds to the 25th percentile of the sample data, and $q_{75}$ corresponds 75th percentile of the sample data ^37^.

Prior studies indicated significant structural remodeling of VTE after seven days^38,39^. Figure 4 depicts the histological composition of VTE less than and greater than seven days old. The thrombus age was designated based on the time between the onset of patient symptoms and the mechanical thrombectomy procedure. Wilcoxon ranked sum tests indicated that thrombi one or more weeks old were composed of more collagen (*p* = 0.03) and fewer erythrocytes (*p* = 0.02) compared to acute specimen. No changes were observed in the fraction of fibrin (*p* = 0.19) or platelets (*p* = 1.00) with thrombus age.

### Immunofluorescent analysis of thrombus

**Figure 5:**
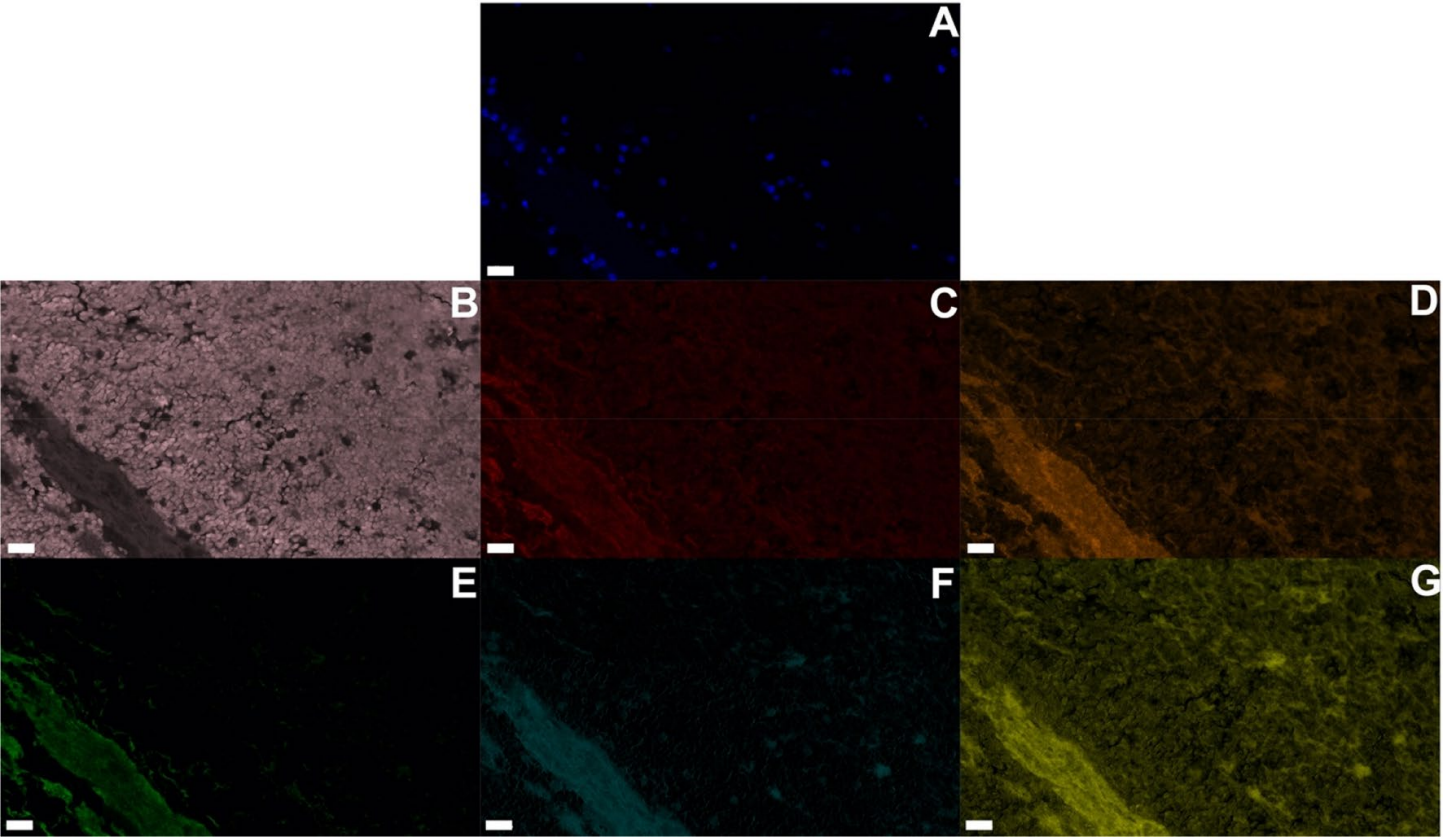
Representative multiplexed immunofluorescent images collected from VTE sample. (**A**) DAPI (nucleated cells) in blue, (**B**) erythrocytes (pink), (**C**) CD61 (platelets, red), (**D**) collagen, types i, ii, and iii (orange), (**E**) fibrin (green), (**F**) PAI-1 (cyan), and (**G**) VEGR-1 (yellow). The scale bar in the lower left corner corresponds to a 20 µm distance (40 × magnification). Antibody information can be found in Supplemental Table 2. A total of eight different antibodies were used for analysis, with five shown here. Autofluorescence was used to visualize erythrocytes. No cross reactivity was observed for antibodies as assessed with human tonsil control samples.

**Figure 6:**
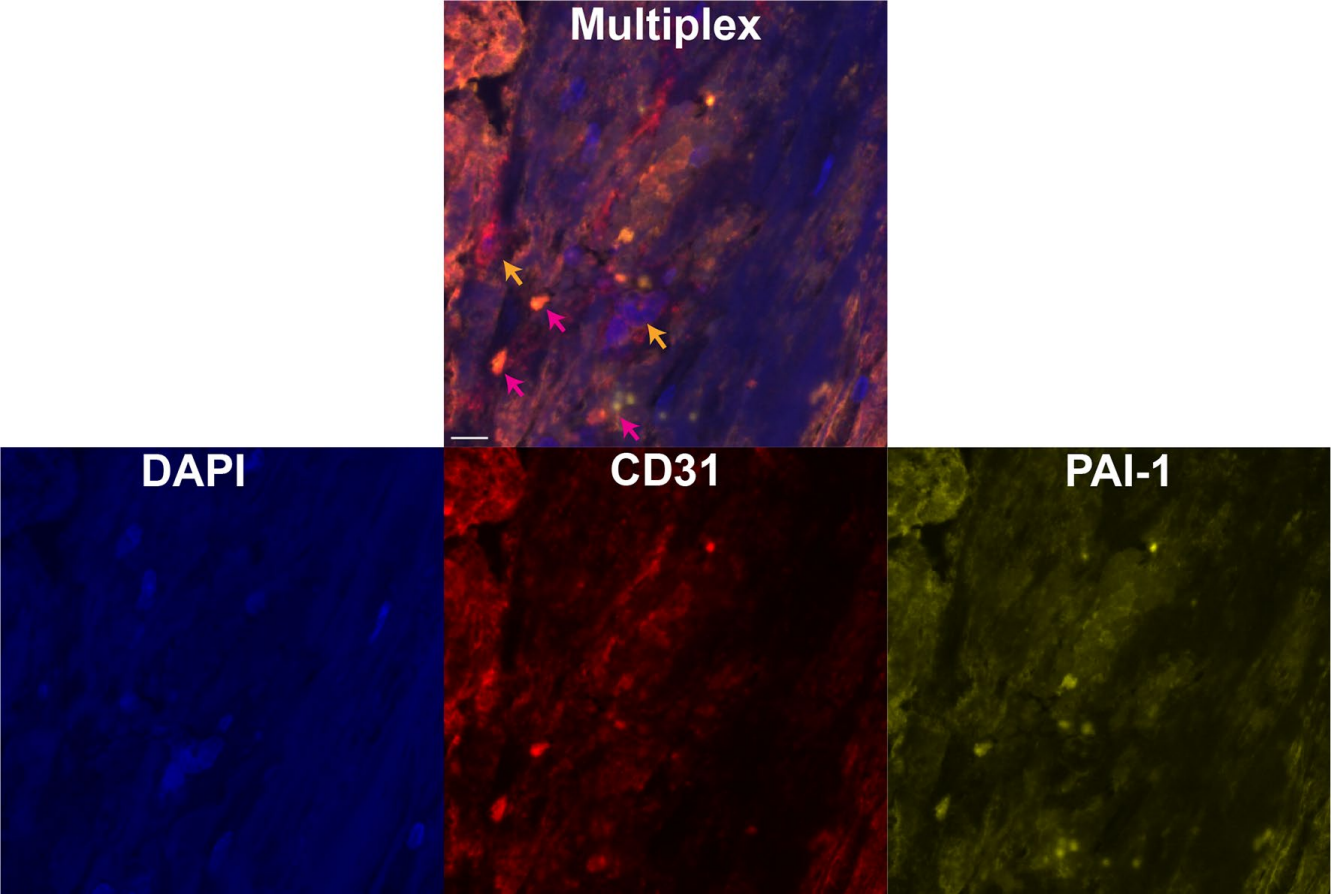
(Top) Representative multiplexed thrombus image indicating nucleated cells assessed with DAPI (blue), PAI-1 (yellow) and CD31 (red). Orange arrows indicate representative locations for cells associated with revascularization (co-expression of DAPI and CD31). Magenta arrows indicate representative areas for co-expression of PAI-1 and CD31. The white bar is a 10 µm distance (40** × **magnification). Multiplex IHC staining was performed on 5 micron FFPE tissue sections using the Opal 7-Color Manual IHC Kit (NEL861001KT; Akoya Biosciences) following the manufacturer instructions. All slides were counterstained with Spectral DAPI (1:10, Akoya Biosciences) nuclear stain and mounted with Slowfade Diamond Antifade mountant (Thermo Fisher Scientific). The slides were then scanned using the Vectra Polaris whole slides scanner (Akoya Biosciences), the regions of interest were selected using the Phenochart software (Akoya Biosciences) and analyzed using Inform software (Akoya Biosciences). Antibody information can be found in Supplemental Table 2. The individual channels for each components are shown in the lower three panels.

Figure 5 depicts representative immunofluorescence staining and imaging to determine the relative position of thrombus components. The extracellular components collagen (types i, ii, iii) and fibrin were observed in close proximity and in regions devoid of erythrocytes. Nucleated cells were observed interspersed within packs of erythrocytes (Supplemental Fig. 1). Possible vascular structure was indicated by regions intersected by vascular endothelial growth factor receptor 1 (VEGFR-1), fibrin, and platelets, and bordered by nucleated cells (Supplemental Fig. 2). Another marker of neovascularization, cluster of differentiation 31 (CD31) was observed within/adjacent to DAPI (nucleated cells). True vascularization, nominally indicated by tubular structures for CD31 positive cells^40^, was not observed though appeared to be in the process of organizing for future recanalization. Plasminogen activator inhibitor-1 (PAI-1) inhibits the fibrinolytic action of rt-PA^41^, and was observed in samples. The inhibitor was co-located or in close proximity to CD31 positive nucleated cells (Fig. 6), fibrin, platelets, and collagen (Fig. 5). Other inhibitors of the fibrinolytic process, including the alpha 2-antiplasmin^42^ and TAFI^43^ were also observed in regions rich with nucleated cells (Supplemental Fig. 3).

### Thrombus stiffness

The elastic moduli of ex vivo samples embedded in agarose varied from 53 to 122 kPa (N = 12 samples, see Supplemental Fig. 4). The elastic modulus of analyzed sample was assessed ex vivo using ultrasound shear wave elastography^44^ in three regions-of-interest to account for sample heterogeneity (see Supplemental Fig. 3). Spearman nonparametric correlations were conducted between the elastic modulus and each histological marker (e.g. concentration of red blood cells, fibrin, collagen, or platelets, N = 12)^45^. No significant correlations were observed between the elastic modulus of the sample and the concentration of thrombus elements. Note that histology was conducted on up to three separate sections for each sample to account for sample heterogeneity. Additionally, no correlation was observed between the elastic modulus of the sample and its susceptibility to rt-PA.

### Thrombus magnetic susceptibility

**Figure 7:**
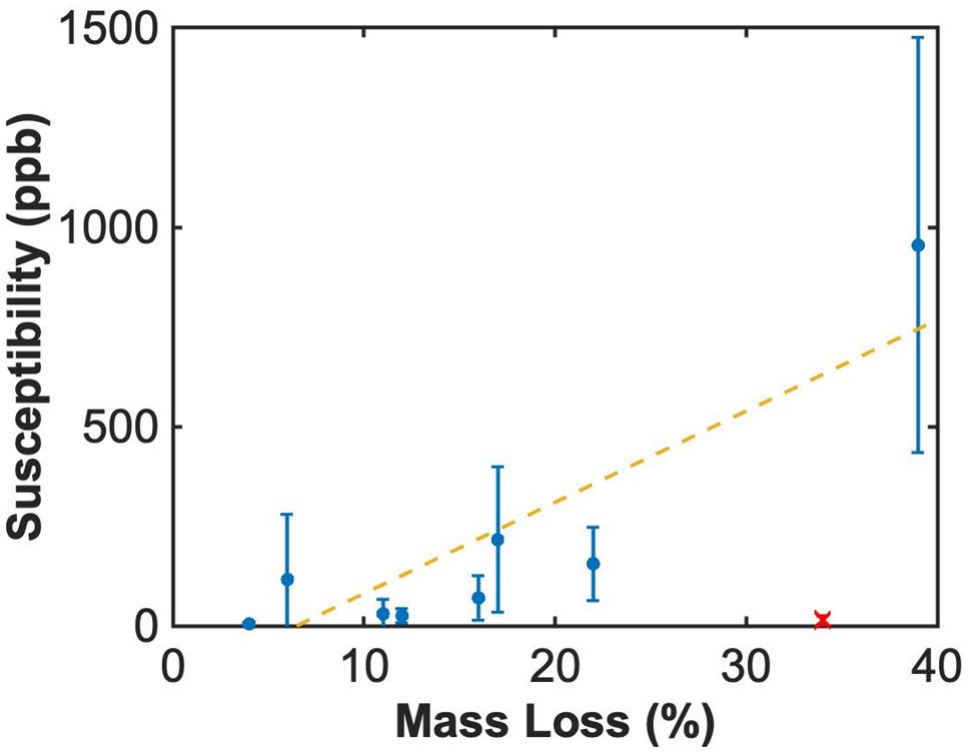
Comparison of sample thrombolytic susceptibility (mass loss) and magnetic susceptibility (QSM analysis). Error bars represent the range of positive QSM values observed within the sample. Separate segments of each specimen were subjected to the clot mass loss assay and imaging analysis. One sample was highly heterogenous (red cross), and gross observation indicated strong variation between the portion of that specimen that was subjected to rt-PA and the portion subjected to QSM analysis. The dashed yellow line is a least-squares best fit to the data. A significant correlation was observed between the thrombolytic and magnetic susceptibility of the sample (Spearman’s rho = 0.90, * p* < 0.01, N = 8 samples) The heterogenous sample (red cross) was excluded from analysis.

The findings in this study and others indicate higher erythrocyte content for acute thrombi relative to chronic disease. Hemoglobin within erythrocytes leads to changes in the local magnetic susceptibility, which can be tracked with a quantitative susceptibility-weighted MRI sequence. The ex vivo specimen collected in this study had heterogenous appearance on susceptibility MRI (Supplemental Fig. 5), potentially due to concentrated zones of high and low iron content. Positive-pixel QSM values indicative of erythrocytic content were tabulated for each sample and correlated with its response to lytic^46^. A significant slope was observed between the magnetic susceptibility of the sample and its disintegration under the action of rt-PA (Fig. 7, Spearman’s rho = 0.9, *p* < 0.01).

## Discussion

A primary goal for this study was to assess the histological properties of venous thromboembolism (VTE). To date, assessment of venous thrombi has been based on animal models or *postmortem* samples^47,48^. Analysis of ex vivo VTE specimen is limited as current approaches treat the thrombus in situ or destroy its structure during removal. The recent advent of venous-specific mechanical thrombectomy devices allowed for the removal of thrombi while still preserving the overall structure^33,35,49^.

Fibrin and erythrocytes were the primary thrombus components observed in these specimen, consistent with the observations of other studies^35^. Compared to a recent investigation of venous thrombus^51^, the prevalence of fibrin (~ 53% vs. 35%) and platelets (~ 30% compared to 4%) were increased and erythrocytes decreased (~ 35% vs. 63%). There may be multiple reasons for the discrepancy between these two studies. Here, the thrombus cross section was analyzed with immunohistochemistry, whereas only subsections of the thrombus were analyzed using scanning electron microscopy in the prior study. Samples here were obtained via venous-specific, minimally-invasive mechanical thrombectomy devices, which may not extract structures adherent to the vessel wall. Open thrombectomy was performed for retrieval of other samples. Regardless, the differences observed indicate a diverse range of VTE composition.

Interestingly, platelets were prevalent within the VTE samples examined here. The standard convention is that venous thrombi are platelet poor, and primarily comprised of erythrocytes entangled in fibrin (i.e. a “red” thrombus)^51^. In contrast, fibrin is through to form as a result of platelet aggregation in arterial thrombi, resulting in minimal erythrocyte content (i.e. “white” thrombus). Platelets contribute to retraction of the thrombus^52^, which is associated with reduced rt-PA susceptibility^53^. Even treatments that do not require intervention may be altered by the prevalence of platelets, indicating that antiplatelet medications typically reserved for arterial thrombi may be appropriate for a subset of VTE^54^. Regardless, these findings indicate there may be less of a distinction between arterial and venous thrombi.

Thrombus age was an important factor in the histological properties of the thrombus, including a reduction in erythrocytes for chronic disease. Prior findings indicate erythrocytes are damaged and removed as the thrombus ages^55^, consistent with the findings here. The shape of erythrocytes vary based on their environment^56^, with the polyhedrocytes shape being the most prevalent shape found in venous thrombi. Further studies are needed to assess the precise form of erythrocytes for the samples collected here.

The extracellular structure of chronic samples was also changed via increases in collagen deposition. The infiltration of fibroblasts into the thrombus is the primary cause of collagen deposition. The process occurs over the course of several days^57,58^, consistent with the lack of observed collagen for acute samples. A hypothesized mechanism of lytic resistance for chronic thrombi is a change in extracellular structure^59,60^. Because of its fibrin-specific proteolysis by plasmin^61^, rt-PA is not effective for collagenous structures. Fibrin was more prevalent than collagen even in chronic samples (on average ~ 60% of thrombus area for fibrin vs. 5% for collagen, Fig. 4), in agreement with another recent analysis of VTE^35^. The method of specimen extraction may contribute to the degree of collagen observed in samples. Fibroblasts are recruited to the thrombus through vessel-wall derived factors, and collagen elements adherent to the vascular wall may not have been retrieved. Nevertheless, these findings suggest fibrin is a primary component of chronic thrombus, and collagen deposition constitutes a secondary effect to the resistance of chronic thrombi. Lytic may therefore still be an effective method for recanalization provided it can be distributed throughout the chronic thrombus extent.

Indicators of neovascularization were observed in some but not all samples. The presence of new vasculature is attributed to remodeling of the venous wall^38^, and is located near the thrombus/vessel interface. Multiple inhibitors to fibrinolysis were observed. Plasminogen activator inhibitor 1 (PAI-1) was observed in samples, a protease inhibitor that acts to quench rt-PA activity and halt fibrinolysis, and may be a key factor in VTE lytic resistance. A subset of nucleated cells within the samples were co-located with PAI-1, though the precise cell type was undetermined. Prior studies indicate that PAI-1 is co-located with neovascularization^62–65^. Markers of neovascularization including VEGFR-1 and CD31 were observed in a subset of samples, though definitive vascular structure could not be confirmed. Leukocytes in the form of macrophages also produce PAI-1^65^, though further work is needed to quantify the precise progenitor for these samples. After generation, PAI-1 is retained within platelets prior to activation. The VTE samples examined here had significant platelet content, which may contribute to prevalence of PAI-1 and lytic resistance of samples. It should be noted PAI-1 was observed to be co-located with platelets (see Fig. 5). Inhibitors for PAI-1 are readily available to reestablish fibrinolysis^66^. Here, PAI-1 was investigated as the most important inhibitor at the plasminogen activation level^67^, though other lytic inhibitors also play a role in thrombolysis (e.g. alpha 2-antiplasminogen and TAFI)^68,69^. Indeed, the markers alpha 2-antiplasmin, which forms a high molecular weight complex with plasmin, and TAFI, which acts as a carboxypeptidase to cleave C-terminal lysine residues from partially digested fibrin, were also present in samples (Supplemental Fig. 3)^69^. Alpha 2-antiplasmin also originates from platelets^70^, and TAFI targets activated platelets^43^. Co-expression of platelets with alpha 2-antiplasmin or TAFI was not investigated here, but will be a focus for future studies, along with other inhibitors to the fibrinolytic cascade.

Etiology was also noted to affect the thrombus structure. Less variability in fibrin and erythrocytes was noted in DVT compared to PE (Fig. 1). The concentration of erythrocytes was noted as a marker of thrombus age (Fig. 4). Over 80% of pulmonary emboli originate as deep vein thrombi^5^. Embolized chronic DVT may serve as a nidus for acute clots, thereby creating a more heterogeneous specimen for PE and therefore the observed increased range of erythrocytes compared to DVT samples.

A secondary goal of this study was to determine the relationship between quantitative imaging metrics (i.e. QSM or ultrasound shear wave elastography) and thrombolytic efficacy. Current imaging metrics for VTE rely on characteristics related to perfusion or stiffness, but lack information on the thrombus microenvironment responsible for its response to lytic therapy. Magnetic resonance imaging is well-suited to measuring the microstructure of thrombi^71^, including erythrocyte content. A significant trend was noted between magnetic susceptibility and thrombolysis (Fig. 7). Deoxygenated hemoglobin within erythrocytes has a high magnetic susceptibility^72^, and is the primary source of contrast for QSM in these samples^73^. The correlation between QSM and thrombolysis indicates lytic susceptibility increases with the concentration of erythrocytes within the thrombus. This agrees with the known relationship between thrombus age and lytic susceptibility and with our observation that older thrombi contain fewer erythrocytes compared to acute thrombi. It should be noted the observed correlation was using a sample size of eight (one sample had grossly heterogenous segments used to assess magnetic and thrombolytic susceptibility, and was excluded from the analysis in Fig. 7). Of the analyzed specimen, one had very strong magnetic and thrombolytic susceptibility which may have skewed the correlation. In the absence of this sample, no correlation was observed between the thrombus mass loss and QSM values (Spearman’s rho = 0.71, *p* = 0.09, N = 7). Nevertheless, these findings motivate continued analysis to determine the precise nature of this relationship between QSM pixel values and rt-PA efficacy.

Prior studies have indicated that increased stiffness is a hallmark of chronic thrombi^74,75^, though no trends were observed between thrombus elastic modulus and histological markers or thrombolysis. The indifference of thrombus properties with its stiffness may be a reflection of the sample age. A prior study demonstrated there is no correlation between ultrasound imaging metrics (e.g. elastic modulus) and thrombus composition for samples older than ~ 10 days^74,76^. Sample ages examined here ranged from 1 to 30 days, with an average and standard deviation of 12.2 ± 9.8 days. The average elastic modulus may also not be a good metric because of sample inhomogeneity, though similar degrees of inhomogeneity is observed for clinical scans of DVT^77,78^. Ultrasound images are inherently two-dimensional, and volumetric assessment of the sample stiffness may provide a more applicable comparison to bulk measurements such as mass loss. Because of its inability to penetrate the lung, ultrasound may not be a viable modality to gauge PE in vivo. Regardless, these data motivate the collection of larger sample sizes to further flush out these trends.

There are a number of limitations to this study that limit generalizability of the finding. Samples were acquired in this study over the course of 24 months (~ one sample per month). The findings and trends noted here warrant additional investigation with larger sample sizes. Samples may be altered by the mechanical extraction procedure. Thrombolytic susceptibility of samples was tested using a systemic lytic administration scheme, whereas catheter-infusion of the lytic within the thrombus is a primary treatment scheme for VTE. Biological factors beyond composition such as blood flow may affect the lytic profile in situ compared to ex vivo^79^. Due to the retrospective nature of this study, sample ages were estimated based on the onset of symptoms and may not reflect the true thrombus age given dependence on reliability patient reporting. To limit the time required for data collection and mitigate sample degradation, separate sections of the thrombus were used for histology, imaging, and assessment of lytic susceptibility. Gross inspections were conducted for samples subjected to each type of analysis, though specimen were heterogenous. Due to the limited size of some of the thrombus samples, not all thrombi could be analyzed with our full testing panel (i.e. histological analysis, thrombolysis, QSM, and elastography). Histological analysis was conducted to determine the presence of fibrin and erythrocytes. The precise phenotype of these elements was not assessed, which can affect relevant properties of the thrombus (e.g. lytic susceptibility^50,80^). Histology and immunofluorescence provide semi-quantitative information on the thrombus structure. Future studies will incorporate quantitative metrics of the thrombus proteins via ELISA or Western blot analysis^81^. The human tonsil used to gauge cross reactivity for the immunofluorescence antibodies is a known control for tumor tissue^82^, though the potential for antibody cross reactivity for thrombus is unknown. Observations indicated good localization of signals from antibodies, though potential regions of non-specific binding were observed. Nevertheless, the observed trends between QSM pixel values and lytic response indicates a potential means to prescreen patients for thrombolytic therapy and thus decrease treatment risk.

## Methods

### Thrombus collection

As part of the standard of care, thrombi were collected from patients undergoing mechanical thrombectomy procedures at the University of Chicago Medical Center and the University of Texas Health Science Center between December 2018 and December 2020. Demographics for patient data are shown in Supplemental Table 1. Thrombi were subjected to analysis following local internal review board (IRB) approval and informed consent (University of Chicago IRB #18–0179). Extracted samples were stored in saline solution and processed for analysis within 24 h of collection. A total of 26 thrombi were examined, of which 12 were deep vein thrombi from iliocaval veins, and 14 were pulmonary emboli. Samples were sectioned, with representative subsections being subjected to histology, imaging, or lytic response. Not all samples were large enough to conduct each set of analyses (26 histology samples, 9 thrombolysis samples, 9 QSM samples, 12 elastography samples).

### Assessment of clot structure via histology

Histology was used for qualitative analysis of the sample structure. Immediately after collection, thrombi were sectioned to ~ 3 mm thickness and submerged in 10% formalin for 24 h. After fixation, thrombus sections were submitted to the University of Chicago Human Tissue Resource Center for paraffin-embedding, sectioning (5 μm thickness), and histochemical staining. Three stains were used: Hematoxylin & Eosin (H&E, Tissue-Tek Prisma H&E Stain Kit #1, Sakura Finetek, USA), Masson’s Trichrome (Trichrome Staining Kit, Hoffmann-La Roche, Switzerland), and anti-CD-61 immunohistochemistry (CD61, Platelet Glycoprotein IIIa, Agilent, USA). Three sections were analyzed for each sample to gauge heterogeneity.

Stained specimen were scanned at 20 × magnification using a ScanScope XT scanner (Leica Biosystems, Germany). Digitized slides were viewed using ImageScope software (Leica Biosystems, Germany), and were analyzed using the Positive Pixel Count algorithm (Leica Biosystems, Germany) to identify red blood cells and fibrin in H&E stains, collagen in Masson’s Trichrome stains, and platelets in CD61 stains^83^. The number of pixels that fell within the designated color parameters (i.e. thrombus component) were quantified and binned into intensity ranges: negative, weak positive, positive, or strong positive. Color thresholds were evaluated and accepted by a board-approved pathologist, as depicted in Supplemental Fig. 5.

Four samples were also subjected to immunofluorescent analysis to quantitatively assess thrombus components. Fluorescent antibodies were used to examine the co-location of fibrin (MABS2155, Millipore Sigma, USA), collagen (Maine medical center research institute, USA), platelets (CD-61, M075301-2, Agilent, USA), vascular endothelial growth factor (VEGFR-1, ab32152, abcam, USA), plasminogen-activator inhibitor-1 (PAI-1, ab125687, abcam, USA), alpha 2-antiplasmin (Hp001885, Atlas Antibodies USA), and thrombin-activatable fibrinolysis inhibitor (TAFI, MBS2006309, MybioSource Inc, USA). Erythrocytes were examined utilizing their innate autofluorescence and did not require staining. Two samples were stained to gauge the co-expression of PAI-1 and the endothelial cell marker CD31 (ab28364, abcam, USA). All slides were counterstained with Spectral DAPI (1:10, Akoya Biosciences). Samples were stained using an Opal 7-color manual IHC kit (Akoya Biosciences, USA) and were scanned using a Vectra Polaris whole slides scanner (Akoya Biosciences, USA). Additional antibody information can be found in Supplemental Table 2.

### Assessment of thrombolytic susceptibility

Human fresh-frozen type O plasma was obtained from a blood bank (Vitalant, Chicago, IL), thawed and aliquoted in 30 mL increments, and stored at -80℃ before use^84^. Alteplase was obtained as a lyophilized powder (Activase, Genetech, San Francisco, CA, USA), mixed with sterile water to a concentration of 1 mg/mL, and stored at -80℃ before use. Ex vivo thrombi were sectioned into ~ 1 cm segments and were exposed to either plasma and rt-PA (2.68 μg/mL, consistent with pharmacomechanical VTE treatment^85^) or plasma alone (control). To account for the heat-dependent enzymatic activity of rt-PA, thrombus sections were placed in individual latex containers filled with 15 mL of human plasma with or without rt-PA and were submerged in a water tank heated to physiologic temperature (37 ℃). The percent change in mass of the sample was tabulated for each sample. Physical manipulation of the thrombi may affect the mass of the clot. To account for these factors, thrombus lytic susceptibility was reported as:

1 $$M_{residual} = M_{lytic} - M_{control}$$

where $M_{lytic}$ is the percent mass loss for clots exposed to lytic and plasma, and $M_{control}$ is the percent mass loss for clots exposed to plasma alone.

### Preparation of samples for imaging

Samples were embedded in 1.5% w/v low gelling temperature agarose before imaging as follows. The agarose solution was heated in a microwave (700 W power) until clear, transferred to an ultrasonic water bath set to 40 ℃, and was degassed for 30 min. After degassing, the agarose solution was cooled to 37 ℃ and then was poured over the thrombus sample. Any bubbles in the agarose were moved outside of the field of view of the thrombus.

### Quantitative susceptibility mapping

Erythrocytes, a primary component of thrombi, can cause significant alterations of the magnetic susceptibility of a sample. Quantitative susceptibility mapping (QSM) is an MRI sequence that maps the sample magnetization. Paramagnetic tissues appear hyperintense in QSM images, and diamagnetic tissues appear hypointense^73^. A clinical 3 Tesla (3T) MR system (Ingenia dStream, Philips Healthcare, Best, The Netherlands) with a body transmit coil and a 16-channel head-and-neck receive coil was used to collect QSM images of the agarose embedded samples. A three-dimensional gradient echo recall sequence was employed to acquire multiple echoes. Utilizing the real and imaginary portions of the received signals, magnetic susceptibility was computed via the morphology enabled dipole inversion (MEDI) pipeline^86,87^. Thrombi were contoured manually to exclude background QSM values. Positive QSM values (i.e. parts per billion, ppb > 0) were tabulated to gauge the concentration of erythrocytes in VTE samples^46^. This technique exploits the paramagnetic nature of hemoglobin while excluding highly diamagnetic tissues.

### Assessment of clot stiffness via ultrasound elastography

Ultrasound elastography was used to assess the stiffness of the agarose-embedded ex vivo samples^44^. Ultrasound images were acquired with a linear array transducer (GE Ultrasound Transducer 9L, 9 MHz nominal frequency) and Logiq E9 scanner (GE Healthcare, Chicago, IL, USA). The scanner exam protocol “Small Parts” was utilized. Based on the approximate depth of the sample, a scan depth of 2 to 4 cm, and 9 MHz frequency setting provided good resolution of the sample. Standard B-mode images were used to visualize the cross section of the thrombus (e.g. orthogonal to its in situ vascular orientation), and a shear wave elastography images were used to map the elastic modulus. Three images (e.g. three measurements of elastic modulus) were recorded, and the average elastic modulus tabulated for each sample. For each section, regions-of-interest ~ 4 mm in diameter were selected to quantify the elastic modulus depending on the diameter of the thrombus (mean diameter 0.7 cm, range 0.3–1.4 cm). To avoid surface waves along the thrombus/agarose interface, regions-of-interest were not acquired near the edge of the sample.

### Statistical analysis

Statistical analysis was performed using the MATLAB Statistical Toolbox (The Mathworks, Natick, MA, USA). Correlations were quantified using the Spearman’s nonparameteric correlation coefficient. Wilcoxon ranked-sum tests were used to compare the composition of thrombi across two age groups and to compare the composition of PE and DVT.

### Ethics declaration

All experimental protocols were approved by the local internal review board and informed consent (University of Chicago IRB #18-0179). All methods were carried out in accordance with the relevant guidelines and regulations. Prior to collecting thrombus samples for analysis, informed consent was obtained. These thrombi were extracted from patients mechanically as part of the standard of care and independent from this study.

## Supplementary Information

Supplementary Information.

## Data Availability

The data generated from this study are available from the authors upon reasonable request.
