# Supplementary material for: Assessment of histological characteristics, imaging markers, and rt-PA susceptibility of ex vivo venous thrombi
Source: Sci Rep. 2021 Nov 23;11:22805. doi: 10.1038/s41598-021-02030-7 (PMC8610976; doi:10.1038/s41598-021-02030-7)
Supplement: Supplementary file 1 — Supplementary Information. [file 41598_2021_2030_MOESM1_ESM.pdf]

Supplementary Material for:

Assessment of histological characteristics, imaging markers, and rt-PA susceptibility of *ex vivo* venous thrombi

Samuel A. Hendley[1], Alexey Dimov[2], Aarushi Bhargava[3], Erin Snoddy[3], Daniel Mansour[3], Rana O. Afifi[4], Geoffrey D. Wool[5], Yuanyuan Zha[6], Steffen Sammet[1,3], Zheng Feng Lu[1,3], Osmanuddin Ahmed[3], Jonathan D. Paul[7], Kenneth B. Bader[1,3*]

1University of Chicago, Committee on Medical Physics, Chicago, IL, 60637, USA

2Weill Cornell Medicine, Department of Radiology, New York, NY, 10021, USA

3University of Chicago, Department of Radiology, Chicago, IL, 60637, USA

4University of Texas at Houston, Department of Cardiothoracic and Vascular Surgery, Houston, TX, 77030, USA

5University of Chicago, Department of Pathology, Chicago, IL 60637, USA

6University of Chicago, The Human Immunological Monitoring Facility, Chicago, IL 60637, USA

7University of Chicago, Department of Medicine, Chicago, IL, 60637, USA

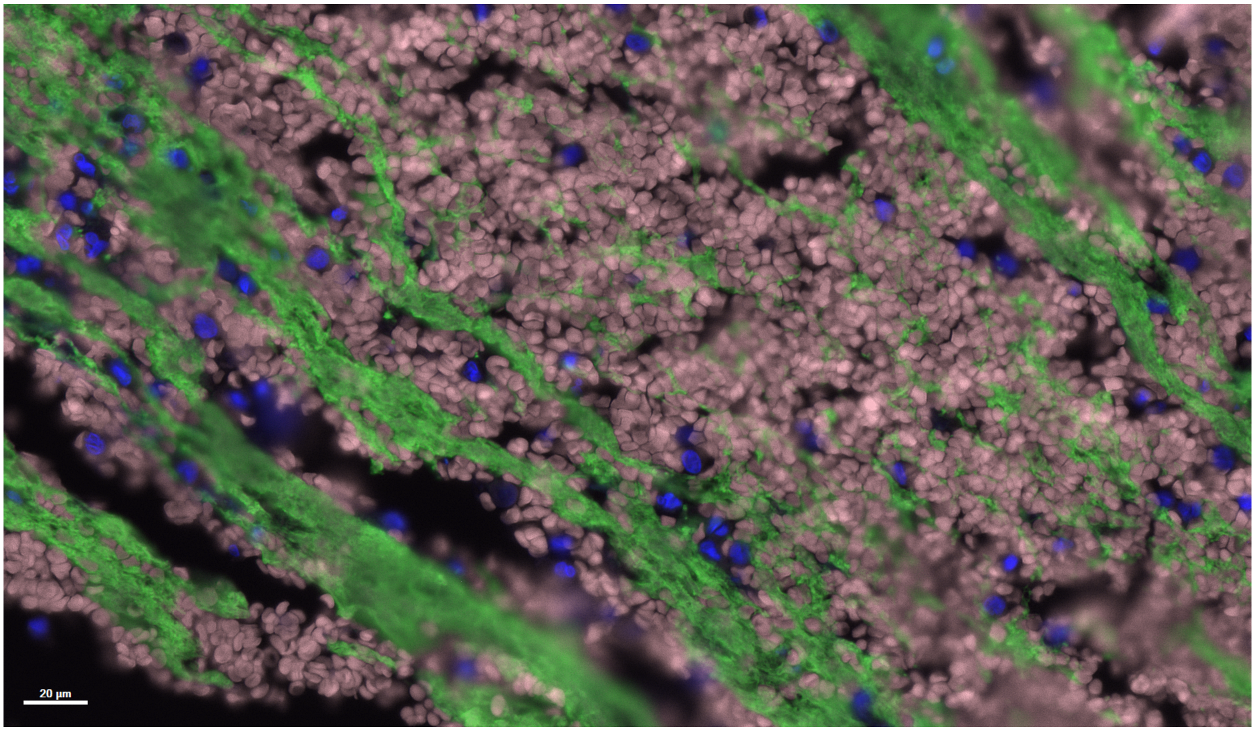

Supplemental Figure 1: Multiplexed immunofluorescent images from VTE sample showing nucleated cells (DAPI in blue) intermixed with erythrocytes (pink) and fibrin (green). The scale bar in the lower left corner corresponds to a 20 µm distance (40x magnification). Antibody information can be found in Supplemental Table 2.

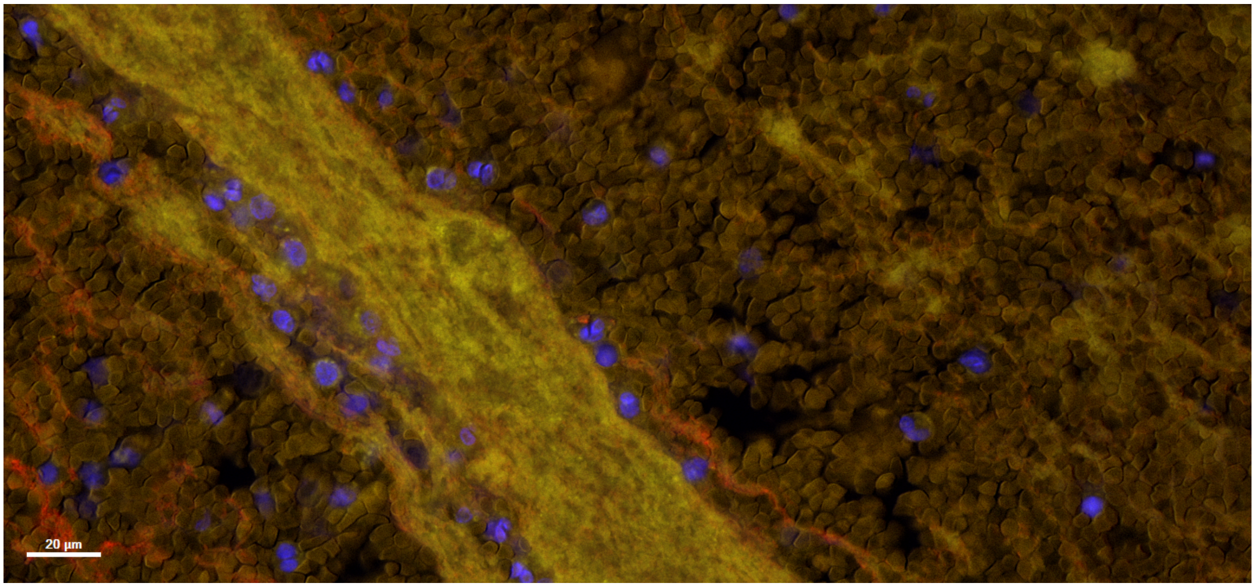

Supplemental Figure 2: Multiplexed immunofluorescent images from VTE sample showing nucleated cells (DAPI in blue) bordering VEGFR-1 (yellow). Platelets (CD61, red) also appear in close proximity to VEGFR-1 (yellow). The scale bar in the lower left corner corresponds to a 20 µm distance (40x magnification). Antibody information can be found in Supplemental Table 2.

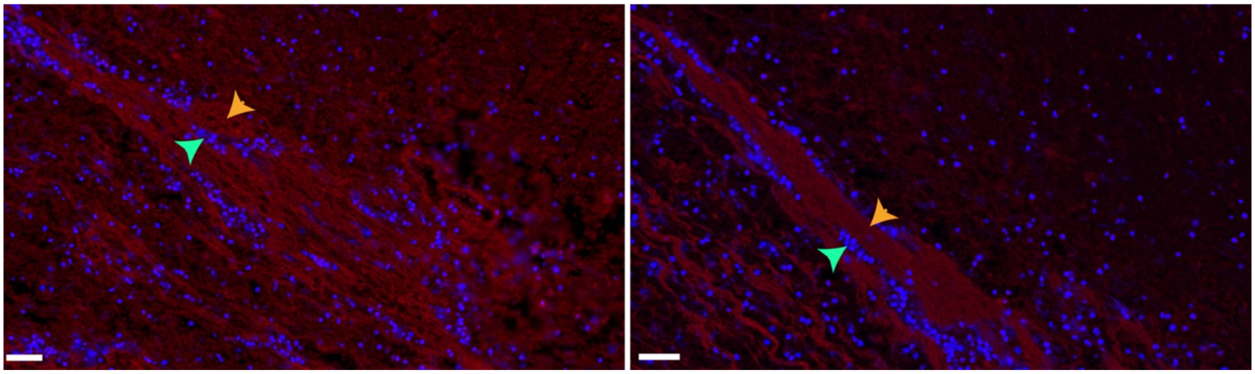

Supplemental Figure 3: Representative multiplexed immunofluorescent images from ex vivo samples highlighting (Left) Alpha 2-antiplasmin (red) boarded by nucleated cells (DAPI in blue), and (Right) Thrombin activatable fibrinolysis inhibitor (TAFI, red) boarded by nucleated cells (DAPI in blue). Green/orange arrows indicate regions where nucleated cells appear to form tubular structures within the respective fibrinolytic inhibitors (alpha 2-antiplasmin or TAFI), suggestive of neovascularization. The scale bar in the lower left corner corresponds to a 50 µm distance (10x magnification). Antibody information can be found in Supplemental Table 2.

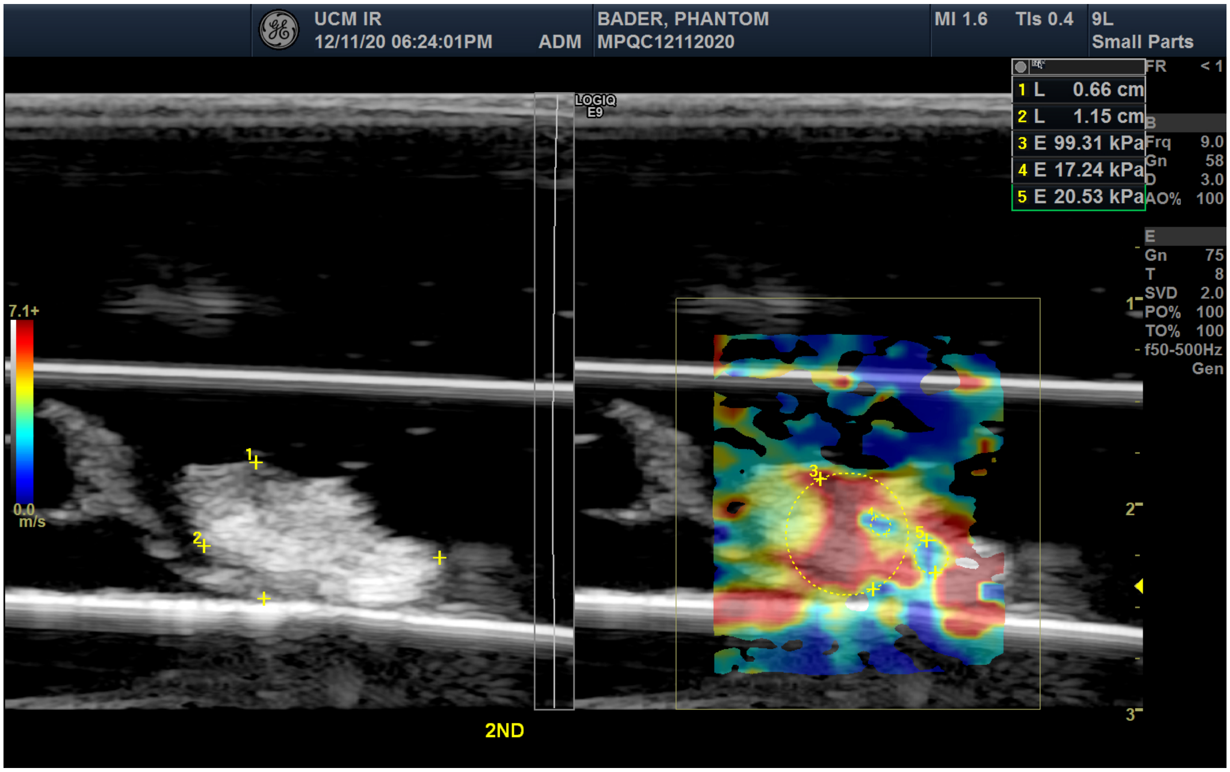

Supplemental Figure 4: Representative B-mode image of *ex vivo* thrombus sample embedded in agarose (left) and image with elasticity map overlay (right). Red indicates high stiffness, and blue indicates low stiffness. Yellow circles circumscribe regions-of-interest for stiffness calculation.

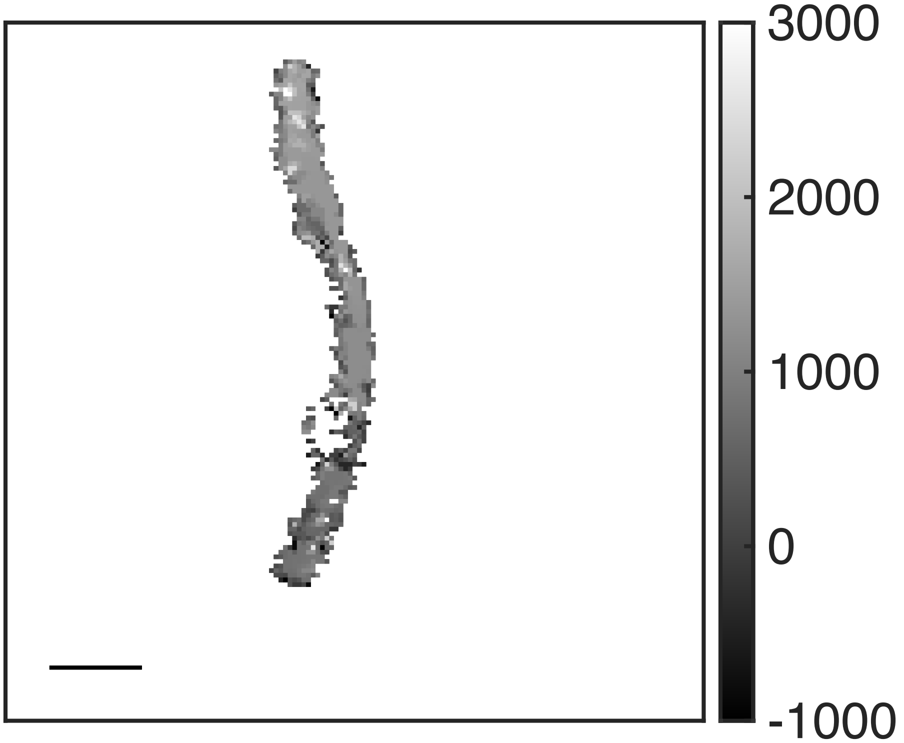

Supplemental Figure 5: Magnetic susceptibility of a representative *ex vivo* thrombus sample embedded in agarose (white background) measured via quantitative susceptibility mapping. The gray colormap represents QSM data values, reported in units of parts per billion (ppb). Bright areas indicate high QSM values, and dark areas indicate low QSM values. Regions represent regions that have high magnetic susceptibility (i.e. erythrocyte rich), whereas dark pixels are have low magnetic susceptibility. The scale bar in the lower left corner corresponds to a distance of 10 mm.

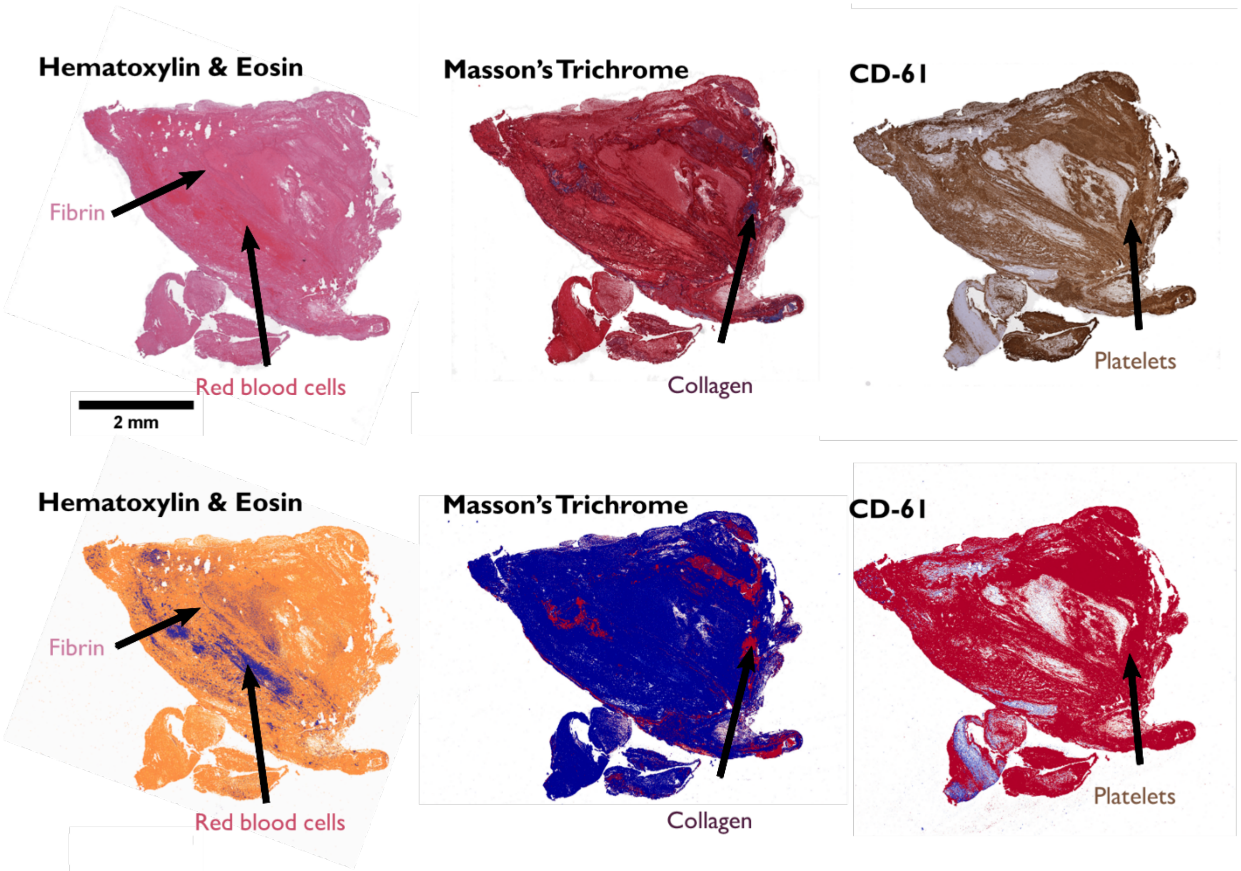

Supplemental Figure 6: Semi-quantitative histological analysis for a representative thrombus sample. The top row indicates sections stained with Hematoxylin & Eosin, Masson's Trichrome, and CD-61. The bottom row indicates the results of the color thresholding algorithm, which counts the number of pixels corresponding to the component of interest.

Supplemental Table 1: Demographic characteristics of VTE patients

**Patient Age (y)**		
Average		Range
57.5		27 – 85
**Race**		
White	Black	Hispanic
5	15	1
**Sex**		
Male		Female
7		14
**Location of Thrombus**		
Deep Vein Thrombosis		Pulmonary Embolism
12		14

Multiplex IHC. Multiplex IHC staining was performed on 5 micron FFPE tissue sections from four thrombus samples using the Opal 7-Color Manual IHC Kit (NEL861001KT; Akoya Biosciences) with the manufacturer's instructions. Briefly, sections were deparaffinized, rehydrated in ethanol gradients, and fixed with 10% neutral buffered formaldehyde. After heat induced epitope retrieval in pH6 citrate buffer, the tissue slides were incubated with primary anti-human antibody, HRP-conjugated secondary antibody, and Opal reagent in a sequential order. The primary antibodies concentrations were optimized as follows: PAI-1 1:50, VEGF-R1 1:50, Fibrin 1:100, Pan Collagen I-III 1:50, CD61 1:40. In a subset of two thrombus tissues, multiplex IHC staining of PAI-1 1:50 and CD31 1:200 or alpha 2-antiplasmin were performed using the same procedure. All slides were counterstained with Spectral DAPI (1:10, Akoya Biosciences) nuclear stain and mounted with

Slowfade Diamond Antifade mountant (Thermo Fisher Scientific). The slides were then scanned using the Vectra Polaris whole slides scanner (Akoya Biosciences), the regions of interest were selected using the Phenochart software (Akoya Biosciences) and analyzed using Inform software (Akoya Biosciences). Details for the antibody and scanning information are indicated in Supplemental Table 2.

Supplemental Table 2: Antibody and scanning information. CD61 and CD31 share the same Opal and filter procedures, but CD31 was used exclusively with PAI-1.

Primary antibody	Vendor/ Catalog Number	Host	Clonality/ Clone Number	Antibody dilution	Opal Fluorophore (nm)
PAI-1	Abcam/ ab125687	Mouse	Monoclonal IgG1 [1D5]	1:50	480
VEGFR-1	Abcam/ ab32152	Rabbit	Monoclonal IgG [Y103]	1:50	570
Fibrin	Millipore Sigma/ MABS2155	Mouse	Monoclonal IgG1 [59D8]	1:100	520
Pan collagen I-III	Main Medical Center Research Institute/Vli42	Rabbit	Monoclonal IgG [Vli42]	1:50	620
Platelets/CD61	Agilent/M075301-2	Mouse	Monoclonal IgG1 [Y2/51]	1:40	690
Endothelial cell marker/CD31	Abcam/ab28364	Rabbit	Polyclonal IgG	1:200	690
Alpha 2-antiplasmin	Atlas Antibodies/Hp001885	Rabbit	Polyclonal IgG	1:200	690
Thrombin-activatable fibrinolysis inhibitor	MybioSource/MBS2006309	Rabbit	Polyclonal	1:25	690
